# Modeling and Generating Extreme Fluctuations in Time Series with a Multilayer Linear Response Model

**DOI:** 10.3390/e27080823

**Published:** 2025-08-03

**Authors:** Yusuke Naritomi, Tetsuya Takaishi, Takanori Adachi

**Affiliations:** 1 Graduate School of Management, Tokyo Metropolitan University, 18F Marunouchi Eiraku Building, 1-4-1 Marunouchi, Chiyoda-ku, Tokyo 100-0005, Japan; taka.adachi@tmu.ac.jp; 2 Department of Liberal Arts, Hiroshima University of Economics, 5-37-1 Gion, Asaminami-ku, Hiroshima 731-0192, Japan; tt-taka@hue.ac.jp

**Keywords:** linear response theory, data generation, heavy-tailed distribution, anomalous dynamics, complex system, statistical physics, financial time series, synthetic data, extreme fluctuation

## Abstract

A multilayer linear response model (MLRM) is proposed to generate time-series data based on linear response theory. The proposed MLRM is designed to generate data for anomalous dynamics by extending the conventional single-layer linear response model (SLRM) into multiple layers. While the SLRM is a linear equation with respect to external forces, the MLRM introduces nonlinear interactions, enabling the generation of a wider range of dynamics. The MLRM is applicable to various fields, such as finance, as it does not rely on machine learning techniques and maintains interpretability. We investigated whether the MLRM could generate anomalous dynamics, such as those observed during the coronavirus disease 2019 (COVID-19) pandemic, using pre-pandemic data. Furthermore, an analysis of the log returns and realized volatility derived from the MLRM-generated data demonstrated that both exhibited heavy-tailed characteristics, consistent with empirical observations. These results indicate that the MLRM can effectively reproduce the extreme fluctuations and tail behavior seen during high-volatility periods.

## 1. Introduction

Recently, remarkable progress has been made in data generation technology, especially in the text and image generation fields. In addition, attempts have been made in the financial sector to generate financial time-series data, e.g., stock prices, with numerous studies utilizing generative adversarial networks (GANs) [1] for this purpose. For example, Ref. [2] proposed Quant GAN, which comprises generator and discriminator functions using temporal convolutional networks (TCNs) [3] to capture long-range dependencies, e.g., the presence of volatility clusters. Similarly, Ref. [4] proposed Stock GAN, based on the Wasserstein GAN (WGAN) [5,6], to address the optimal transport problem [7]. More recently, Ref. [8] proposed Sig-Wasserstein GAN (Sig-WGAN), which combines rough path theory [9] and the expected signature [10] to improve training stability and sample quality. Building on this, Ref. [11] extended the Sig-WGAN framework to the conditional setting and proposed a conditional Sig-WGAN for modeling the distribution of future time series given past observations. Moreover, Ref. [12] proposed a conditional GAN-based market simulator that reacts to experimental agent behavior, enabling realistic and responsive market simulations to evaluate trading strategies. In addition, Ref. [13] introduced Fin-GAN, a GAN-based model for financial time series forecasting, which outperforms traditional methods like LSTM [14] and ARIMA [15] in both predictive accuracy and classification performance.

Recently, diffusion models [16] have been developed rapidly and employed for the generation of time-series data. For example, Ref. [17] introduced a two-way variational autoencoder, $D^{3}$VAE, with diffusion, denoising, and disentanglement, to address multivariate time-series forecasting problems. The $D^{3}$VAE model improves denoising diffusion and score matching by treating discrete observations as continuous values in the function space. In the context of missing value imputation, which is critical for practical financial applications, Ref. [18] proposed a conditional score-based diffusion model (CSDI). Furthermore, Ref. [19] introduced a structured state space diffusion (SSSD) model, which is particularly effective at capturing long-term dependencies in time-series data. More recently, Ref. [20] proposed a conditional diffusion model as a denoiser for financial time-series data, demonstrating an improved signal-to-noise ratio, return predictability, and trading performance compared to existing methods such as denoising autoencoders and moving averages. In addition, Ref. [21] proposed FTS-Diffusion, a diffusion-based generative framework that specifically models the irregular and scale-invariant patterns of financial time series, achieving high fidelity in synthetic data generation and significant improvements in downstream forecasting tasks.

In parallel with these developments, transformer-based architectures have attracted significant attention in financial time-series forecasting and generation. Unlike traditional RNNs and LSTMs, transformers leverage self-attention to capture long-term dependencies and complex patterns, and they have been shown to outperform conventional deep learning approaches for stock prediction tasks [22,23,24]. These advances highlight the growing importance of and attention-based models in financial and commodity time-series modeling.

However, there are several problems in applying machine learning models, including GANs, diffusion models, and transformer-based architectures, to financial time-series data. First, the numerous parameters in these models make them “black boxes,” making it difficult to explain the causal relationship between inputs and outputs. Second, there is a significant risk of overfitting to the training data, where even subtle differences between the input and training data can lead to substantially different outputs. In addition, existing machine learning approaches, including RNNs, LSTMs, and even transformer-based models, face fundamental limitations in financial contexts: traditional linear and parametric models struggle to capture the nonlinear and non-stationary dynamics of financial time series, while deep learning models often face difficulty modeling long-term dependencies and effectively selecting relevant features from high-dimensional or noisy inputs. This is particularly true due to the lack of explicit feature-level attention mechanisms, which can be crucial for accurate multivariate time-series forecasting [25]. Recently, it has been reported that explainable machine learning models, such as LightGBM, can achieve high prediction accuracy in financial time-series tasks by leveraging their feature importance and transparency [26]. Linear response theory (LRT), a physics-based method grounded in the framework of nonequilibrium statistical mechanics, offers a potential solution to these problems [27,28]. In LRT, the input is treated as an external force (i.e., a cause), and the output is the generated data (i.e., the result); this framework enables the system to produce robust responses to external force, as the coupling of these causal relationships is interpreted as a second-order fluctuation in the system.

In a related study applying LRT to finance, Ref. [29] analyzed NASDAQ data from 862 stocks and identified log-return as the most appropriate conjugate variable, demonstrating strong agreement between theoretical predictions and empirical average responses. Their study also examined the semi-strong efficiency of various financial markets, including Forex (EUR/USD, USD/HKD), Bitcoin, commodities (oil and gold), and the S&P500. Despite being a physical model, LRT accurately captures average price reactions to market events, providing valuable insights into market dynamics [30].

Previously, we proposed a linear response model (LRM) [31] to generate time-series data based on LRT. The LRM is derived under the assumption that the distribution of the time-series data follows an energy-based model (EBM) [32], with the energy function approximated up to the second-order around the mean. A key advantage of the LRM is that it avoids overfitting because model training is not required, and it avoids the black box issue because the causal relationships are explicitly known. In addition, the LRM offers several other benefits, e.g., very low computational costs. The LRM can be applied to financial time-series to generate data with fluctuations of various magnitudes; however, it is difficult to reproduce dynamics involving typically large fluctuations, e.g., those caused by COVID-19. While the LRM is primarily designed as a data generation method, there has been increasing attention in recent years to the detection and modeling of anomalous dynamics, which are often associated with extreme events. In this context, machine learning models such as normalizing flows have attracted considerable interest, and a variety of approaches have been explored to better capture rare anomalies and extreme dynamics in time-series data [33].

In order to overcome this issue, we propose the multilayer LRM (MLRM) to generate anonymous dynamics. The proposed MLRM is constructed by extending the LRM to multiple layers, which means that the MLRM incorporates the output data of the LRM iteratively. As a result, the fluctuations in the generated time-series data are amplified, thereby producing time-series data with anomalous dynamics. However, the data generated via the proposed MLRM may diverge. To solve this problem, it is necessary to consider methods to suppress divergence. Note that this approach is analogous to renormalization in quantum field theory [34], which is employed to avoid divergence in self-energy calculations.

By applying the proposed MLRM to financial time-series data, we attempted to determine whether large fluctuation dynamics can be generated using the information of fluctuations prior to COVID-19. Furthermore, we calculated the return distribution and the distribution of realized volatility from the data generated via the proposed MLRM. The return distribution exhibited pronounced fat-tailed behavior, with the estimated tail index being significantly smaller than 3 and close to 2, thereby deviating from the so-called inverse cubic law [35,36], and resembling the inverse square law observed during the COVID-19 pandemic and other extreme market periods [37,38]. The realized volatility distribution was well described by an inverse gamma distribution [39,40].

Praetz [41] demonstrated that Student’s *t* distribution provides a more accurate model for financial returns than the normal distribution, as it can accommodate the heavy tails observed in empirical data. Separately, the nonextensive statistical mechanics framework, originally formulated by Tsallis and his colleagues, offers a theoretical foundation for understanding the emergence of power-law distributions in complex systems, including financial markets [42]. In particular, this approach explains the frequent observation of fat-tailed return distributions and the breakdown of conventional Boltzmann–Gibbs statistics in empirical financial data. Our results, which show extremely heavy tails in the return distributions generated via the MLRM, are consistent with the predictions of nonextensive statistics and further support the relevance of this framework for modeling extreme market events, such as those observed during the COVID-19 pandemic.

## 2. Theory

First, in Section 2.1, we explain the theoretical framework of our previously proposed LRM [31] as a generative model for time-series data under external forces. Specifically, we begin by introducing the probability distribution of time-series data using an EBM, and then we incorporate external forces into the energy function to define the probability distribution of time-series data subject to such external forces. By approximating the energy function up to the second order, we demonstrate that time-series generation under external forces can be characterized by the product of the second-order fluctuation (evaluated in the absence of external forces) and the applied external force. Subsequently, in Section 2.2, we extend the LRM described in Section 2.1 to a multilayer framework. In this extension, the external force is treated as a random variable, enabling the generation of time-series data under various external forces. Furthermore, we assume that the fluctuations in data generated under various external forces themselves follow the LRM, and by iteratively applying this procedure, we derive the MLRM. Finally, since the derived MLRM may exhibit divergence under certain conditions, we also discuss methods to suppress such divergence and ensure the stability of the model.

### 2.1. LRM

Here, we review the theory of a single-layer LRM (SLRM) or a simple LRM for the generation of time-series data [31]. Let T:={1,2,3,⋯,N} be the time domain for which we observe the real-valued *M*-dimensional time-series. Then, for each t∈T, the observing values

(1) $$x_{t}:={\left(x_{t,1},x_{t,2},\cdots ,x_{t,M}\right)}^{T}\in R^{M}$$

are considered to be the realization of the random variables $X_{t}:\Omega \to R^{M}$, where Ω is the underlying set of a filtered probability space $\left(\Omega ,F,{\left\{F_{t}\right\}}_{t\in T},P\right)$.

Assume that, for the random variable

(2) $$X:\Omega \to T\times R^{M}\approx R^{N\times M},$$

the following function exists:

(3) $$E_{X}:R^{N\times M}\to R_{+}.$$

This is referred to as an energy function, where the probability density function, $f_{X}$, of X is expressed as follows:

(4) $$\rho_{X}\left(x\right):=\frac{e^{-E_{X}\left(x\right)}}{Z_{0}}.$$

Here, $x:={\left(x_{1},x_{2},\cdots ,x_{N}\right)}^{T}\in R^{N\times M}$, and we have the following:

(5) $$Z_{0}:=\int_{R^{N\times M}}dx\;e^{-E_{X}\left(x\right)}.$$

In other words, our description is based on the EBM introduced by [32].

In addition, the notation ${\langle \cdots \rangle }_{0}$ represents the ensemble average, i.e., for a function, $A_{i}:R^{N\times M}\to R,$ we obtain the following for i=1,⋯,N×M:

(6) $$\begin{array}{c} {\langle A_{i}\rangle }_{0}:=\int_{R^{N\times M}}d\;xA_{i}\left(x\right)\rho_{X}\left(x\right). \end{array}$$

Next, we consider the Taylor expansion of (4) around a mean value:

(7) $$\mu_{t}:={\langle x_{t}\rangle }_{0}.$$

As a result, if we assume that $E_{X}$ can be approximated up to the second-order term of the displacement random vector $\Delta x_{t}:=x_{t}-\mu_{t}$, we obtain

(8) $$E_{X}\left(x\right)\approx E_{X}\left(\mu \right)+\sum_{t=1}^{N}g_{t}\cdot \Delta x_{t}+\frac{1}{2}\sum_{s,t=1}^{N}\Delta x_{s}^{T}H_{st}\Delta x_{t},$$

where

$$\begin{array}{cc} \; & g_{t}:={\left.\frac{\partial E_{X}}{\partial x_{t}}\right|}_{x_{t}=\mu_{t}}\in R^{M}\\ \; & ={\left.{\left(\frac{\partial E_{X}}{\partial x_{t,1}},\cdots ,\frac{\partial E_{X}}{\partial x_{t,M}}\right)}^{T}\right|}_{x_{t}=\mu_{t}} \end{array}$$

is the gradient vector, and

(9) $$\begin{array}{cc} \; & H_{st}:={\left.\frac{\partial^{2}E_{X}}{\partial x_{s}\partial x_{t}}\right|}_{x_{s}=\mu_{s},x_{t}=\mu_{t}}\in R^{M\times M}\\ \; & ={\left.\left(\begin{array}{ccc} \frac{\partial^{2}E_{X}}{\partial x_{s,1}\partial x_{t,1}}\; & \cdots \; & \frac{\partial^{2}E_{X}}{\partial x_{s,M}\partial x_{t,1}}\\ \vdots \; & \ddots \; & \vdots \\ \frac{\partial^{2}E_{X}}{\partial x_{s,1}\partial x_{t,M}}\; & \cdots \; & \frac{\partial^{2}E_{X}}{\partial x_{s,M}\partial x_{t,M}} \end{array}\right)\right|}_{x_{s}=\mu_{s},x_{t}=\mu_{t}} \end{array}$$

is the Hessian matrix. Under the assumption that the gradient is zero ($g_{t}=0$) at the mean value, (8) becomes the following:

(10) $$E_{X}\left(x\right)\approx E_{X}\left(\mu \right)+\frac{1}{2}\sum_{s,t=1}^{N}\Delta x_{s}^{T}H_{st}\Delta x_{t}.$$

In addition, the first term, $E_{X}\left(\mu \right)$, in (10) is a constant. Thus, the numerator and denominator in (4) cancel each other out, and finally, with the substitution of (10) with (4), (4) is rewritten as follows:

(11) $$\rho_{X}\left(x\right)=\frac{1}{Z_{0}^{\prime }}e^{-\frac{1}{2}\sum_{s,t=1}^{N}\Delta x_{s}^{T}H_{st}\Delta x_{t}},$$

where

(12) $$Z_{0}^{\prime }:=\int_{R^{N\times M}}dx\;e^{-\frac{1}{2}\sum_{s,t=1}^{N}\Delta x_{s}^{T}H_{st}\Delta x_{t}}.$$

Note that (11) takes the form of a multidimensional Gaussian distribution; however, it is dependent on both *s* and *t*. In addition, the Hessian matrix is equivalent to the following inverse matrix (refer to Section 1.4 in the literature [27]):

(13) $$H_{st}=C_{st}^{-1},$$

where

(14) $$\begin{array}{c} C_{st}:={\langle \Delta x_{s}\Delta x_{t}^{T}\rangle }_{0}\in R^{M\times M}. \end{array}$$

Next, assume that an external force vector $f_{t}\in R^{M}$ acts on the system, and the external force energy term $-\sum_{t=1}^{N}\Delta x_{t}\cdot f_{t}$ is added to the original energy function $E_{X}$ as follows:

(15) $$E_{X}\left(x\right)-\sum_{t=1}^{N}\Delta x_{t}\cdot f_{t}.$$

Thus, the probability distribution under external forces is expressed as follows:

(16) $$\rho_{X}^{f}\left(x\right):=\frac{1}{Z_{f}^{\prime }}e^{-\frac{1}{2}\sum_{s,t=1}^{N}\Delta x_{s}^{T}C_{st}^{-1}\Delta x_{t}+\sum_{t=1}^{N}\Delta x_{t}\cdot f_{t}},$$

where

(17) $$Z_{f}^{\prime }:=\int_{R^{N\times M}}dx\;e^{-\frac{1}{2}\sum_{s,t=1}^{N}\Delta x_{s}^{T}C_{st}^{-1}\Delta x_{t}+\sum_{t=1}^{N}\Delta x_{t}\cdot f_{t}}.$$

To obtain the response ${\langle \Delta x_{t}\rangle }_{f}$ under external forces, it is sufficient to take the partial derivative of the logarithm of (17) with respect to $f_{t}$, as follows:

(18) $$\begin{array}{cc} {\langle \Delta x_{t}\rangle }_{f} & =\frac{\partial }{\partial f_{t}}\ln Z_{f}^{\prime }\\ \; & =\frac{1}{Z_{f}^{\prime }}\frac{\partial }{\partial f_{t}}Z_{f}^{\prime }\\ \; & =\frac{1}{Z_{f}^{\prime }}\int_{R^{N\times M}}dx\;\Delta x_{t}e^{-\frac{1}{2}\sum_{s,t=1}^{N}\Delta x_{s}^{T}C_{st}^{-1}\Delta x_{t}+\sum_{t=1}^{N}\Delta x_{t}\cdot f_{t}}\\ \; & =\int_{R^{N\times M}}dx\rho_{X}^{f}\left(x\right)\Delta x_{t}. \end{array}$$

The notation ${\langle \cdots \rangle }_{f}$ represents the ensemble average under the external force for i=1,⋯,N×M:

(19) $$\begin{array}{c} {\langle A_{i}\rangle }_{f}:=\int_{R^{N\times M}}d\;xA_{i}\left(x\right)\rho_{X}^{f}\left(x\right). \end{array}$$

To calculate (18), the variable transformation $\Delta x_{t}^{\prime }:=\Delta x_{t}-\sum_{k=1}^{N}C_{tk}f_{k}$ for (17) makes it possible to obtain the following:

(20) $$\begin{array}{cc} Z_{f}^{\prime }\; & =\int_{R^{N\times M}}dx^{\prime }\;e^{-\frac{1}{2}\sum_{s,t=1}^{N}\left(\Delta x_{s}^{\prime T}+\sum_{k=1}^{N}f_{k}^{T}C_{ks}\right)C_{st}^{-1}\left(\Delta x_{t}^{\prime }+\sum_{k=1}^{N}C_{tk}f_{k}^{T}\right)+\sum_{t=1}^{N}\left(\Delta x_{t}^{\prime }+\sum_{s=1}^{N}f_{s}^{T}C_{st}\right)\cdot f_{t}}\\ \; & =\int_{R^{N\times M}}dx^{\prime }\;e^{-\frac{1}{2}\left(\sum_{s,t=1}^{N}\Delta x_{s}^{\prime T}C_{st}^{-1}\Delta x_{t}^{\prime }+2\sum_{t=1}^{N}\Delta x_{t}^{\prime }\cdot f_{t}+\sum_{s,t=1}^{N}f_{s}^{T}C_{st}f_{t}\right)+\sum_{t=1}^{N}\left(\Delta x_{t}^{\prime }+\sum_{s=1}^{N}f_{s}^{T}C_{st}\right)\cdot f_{t}}\\ \; & =\int_{R^{N\times M}}dx^{\prime }\;e^{-\frac{1}{2}\sum_{s,t=1}^{N}\Delta x_{s}^{\prime T}C_{st}^{-1}\Delta x_{t}^{\prime }+\frac{1}{2}\sum_{s,t=1}^{N}f_{s}^{T}C_{st}f_{t}}\\ \; & =Z_{0}^{\prime }e^{\frac{1}{2}\sum_{s,t=1}^{N}f_{s}^{T}C_{st}f_{t}}. \end{array}$$

In this derivation, we have used the relation $C_{st}=C_{ts}$ and $\sum_{s=1}^{N}C_{ks}C_{st}^{-1}=I_{kt}\in R^{M\times M}$, where $I_{kt}$ is the M×M identity matrix for k=t, and the zero matrix otherwise.

By substituting (20) with (18), we obtain the following:

(21) $${\langle \Delta x_{t}\rangle }_{f}={\langle x_{t}\rangle }_{f}-\mu_{t}=\sum_{s=1}^{N}C_{ts}f_{s}.$$

(21) is the equation of the LRM for time-series data and discrete time. If the time moves continuously in the interval $\left[t_{0},t_{1}\right]$, then (21) can be rewritten as follows:

(22) $${\langle \Delta x(t)\rangle }_{f}=\int_{t_{0}}^{t_{1}}C\left(t,s\right)f\left(s\right)ds,$$

where ${\langle \Delta x(t)\rangle }_{f}$, C(t,s), and f(s) are regarded as ${\langle \Delta x_{t}\rangle }_{f}$, $C_{t,s}$, and $f_{s}$ functions of continuous time, respectively. (22) takes the form of the green function in physics. We refer to $C_{st}$ or C(s,t) as a two-time covariance matrix or function. In addition, (22) can be interpreted as the function f that minimizes the following variational function:

(23) $$\begin{array}{cc} \Phi [f]\; & :=\int_{t_{0}}^{t_{1}}dt{\langle \Delta x(t)\rangle }_{f}\cdot f\left(t\right)\\ \; & -\frac{1}{2}\int_{t_{0}}^{t_{1}}dt\int_{t_{0}}^{t_{1}}ds\;f{\left(t\right)}^{T}C\left(t,s\right)f\left(s\right). \end{array}$$

With a return to the discrete time version, (21) can be rewritten as follows:

(24) $${\langle \Delta x\rangle }_{f}={\langle x\rangle }_{f}-\mu =Cf,$$

where

$$\begin{array}{cc} \; & {\langle x\rangle }_{f}:=\left(\begin{array}{c} {\langle x_{1}\rangle }_{f}\\ {\langle x_{2}\rangle }_{f}\\ \vdots \\ {\langle x_{N}\rangle }_{f} \end{array}\right),\mu :=\left(\begin{array}{c} \mu_{1}\\ \mu_{2}\\ \vdots \\ \mu_{N} \end{array}\right),\\ \; & f:=\left(\begin{array}{c} f_{1}\\ f_{2}\\ \vdots \\ f_{N} \end{array}\right),C:=\left(\begin{array}{cccc} C_{11} & C_{12} & \cdots & C_{1N}\\ C_{21} & C_{22} & \cdots & C_{2N}\\ \vdots & \vdots & \ddots & \vdots \\ C_{N1} & C_{N2} & \cdots & C_{NN} \end{array}\right). \end{array}$$

**Figure 1:**
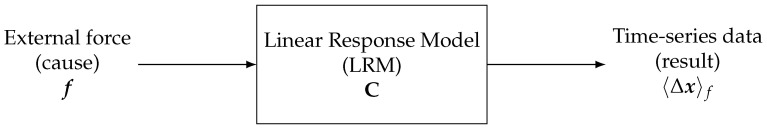
Data generation using the LRM.

Here, ${\langle \Delta x\rangle }_{f}\in R^{L}$, $C\in R^{L\times L}$, and $f\in R^{L}$ for L:=N×M are the vector of ${\langle \Delta x_{t}\rangle }_{f}$, the matrix of the expanded versions in the time direction of $C_{st}$, and the vector of $f_{t}$, respectively. Figure 1 shows a schematic representation of the LRM. Here, the input is treated as an external force (i.e., a cause), and the output is the generated data (i.e., the result); furthermore, the coupling of these causal relationships is considered a second-order fluctuation of the system.

### 2.2. MLRM

In the following section, we extend the conventional LRM to multiple layer in order to construct the proposed MLRM.

First, let the external force f be the observing values of a random variable, $F:\Omega \to R^{L}$. The probability density function of F is represented as $\rho_{F}\left(f\right)$. Then, ${\langle \Delta x\rangle }_{f}$ becomes a conditional expectation of Δx, given F=f. Here, ${\langle \Delta x\rangle }_{f,n}$ denotes the data generated via the *n*-layer LRM. Then, (24) of the SLRM can be expressed as follows:

(25) $${\langle \Delta x\rangle }_{f,1}:={\langle x\rangle }_{f,1}-{\langle x\rangle }_{f,0}=C_{0}f,$$

where ${\langle x\rangle }_{f,0}:=\mu$, $C_{0}:=C$.

The two-time covariance matrix $C_{1}$ of the data generated via the SLRM is given as follows:

(26) $$C_{1}:=\langle {\langle \Delta x\rangle }_{f,1}{\langle \Delta x\rangle }_{f,1}^{T}\rangle =C_{0}\langle ff^{T}\rangle C_{0}^{T}=C_{0}\Sigma C_{0},$$

where the relationship $C_{0}^{T}=C_{0}$ was used, and

(27) $$\begin{array}{c} \Sigma :=\langle ff^{T}\rangle :=\int_{R^{N\times M}}d\;f\left(ff^{T}\right)\rho_{F}\left(f\right). \end{array}$$

Under the assumption that the data generated via the first layer of the LRM follow the second layer of the LRM, the displacement of the second layer of the LRM at that time is denoted as ${\langle \Delta x\rangle }_{f,2}$, which is expressed as follows:

(28) $$\begin{array}{c} {\langle \Delta x\rangle }_{f,2}:={\langle x\rangle }_{f,2}-{\langle x\rangle }_{f,1}=C_{1}f. \end{array}$$

(28) represents the data generation of the two-layer LRM. The equation for the two-layer LRM is expressed as follows:

(29) $$\begin{array}{cc} {\langle x\rangle }_{f,2} & ={\langle x\rangle }_{f,1}+C_{1}f\\ \; & ={\langle x\rangle }_{f,0}+C_{0}f+C_{0}\Sigma C_{0}f\\ \; & =\mu +Cf+C\Sigma Cf. \end{array}$$

By repeating these operations *n* times, we can derive the data generation equation for an *n*-layer LRM (*n*-LRM). Here, the following series is obtained:

(30) $$\begin{array}{cc} {\langle x\rangle }_{f,n} & =\mu +C_{0}f+C_{1}f+\cdots +C_{n-1}f\\ \; & =\mu +\left(C_{0}+C_{1}+\cdots +C_{n-1}\right)f\\ \; & =\mu +\sum_{p=0}^{n-1}C_{p}f, \end{array}$$

where

(31) $$\begin{array}{c} C_{p}:=C_{p-1}\Sigma C_{p-1} \end{array}$$

for p=1,⋯,n−1.

By substituting (31) with (30), we obtain the following:

(32) $$\begin{array}{cc} {\langle x\rangle }_{f,n} & =\mu +\Sigma^{-1}\left(B+B^{2}+B^{4}+\cdots +B^{2^{n-1}}\right)f\\ \; & =\mu +C(n)f, \end{array}$$

where

(33) $$\begin{array}{cc} C(n) & :=\sum_{p=0}^{n-1}C_{p}=\Sigma^{-1}B\left(n\right), \end{array}$$

(34) $$\begin{array}{cc} B(n) & :=B+B^{2}+B^{4}+\cdots +B^{2^{n-1}}, \end{array}$$

(35) $$\begin{array}{cc} B & :=\Sigma C. \end{array}$$

Note that B is generally an asymmetric matrix.

In the following, we consider a case when the number of layers proceeds to infinity. Here, we refer to the resulting model as the *∞*-LRM. In other words, if *n* in (32) is set to infinity, we obtain the following:

(36) $$\begin{array}{cc} {\langle x\rangle }_{f,\infty } & =\mu +\Sigma^{-1}\left(B+B^{2}+B^{4}+\cdots \right)f\\ \; & =\mu +C_{\mathrm{eff}}f, \end{array}$$

where

(37) $$\begin{array}{cc} C_{\mathrm{eff}} & :=C\left(\infty \right)=\Sigma^{-1}B_{\mathrm{eff}}, \end{array}$$

(38) $$\begin{array}{cc} B_{\mathrm{eff}} & :=B\left(\infty \right)=B+B^{2}+B^{4}+\cdots . \end{array}$$

Here, $C_{\mathrm{eff}}$ is the two-time covariance matrix when (32) converges at infinity and becomes the finite values.

The eigenvalue problem for the asymmetric matrix B is solved as follows:

(39) $$\begin{array}{cc} BV_{R} & =V_{R}\bar{\Lambda }, \end{array}$$

(40) $$\begin{array}{cc} V_{L}^{T}B & =\bar{\Lambda }V_{L}^{T}, \end{array}$$

(41) $$\begin{array}{cc} V_{L}V_{R}^{T} & =V_{R}^{T}V_{L}=I. \end{array}$$

Here, $V_{R}:=\left(v_{1}^{R},\cdots ,v_{L}^{R}\right)\in R^{L\times L}$ is a matrix of a column of right eigenvectors $v_{i}^{R}$ for i=1,⋯,L, $V_{L}\in R^{L\times L}$ is a matrix of a column of left eigenvectors $v_{i}^{L}$ for i=1,⋯,L, and $\bar{\Lambda }\in R^{L\times L}$ is a diagonal matrix whose *i*th diagonal element ${\bar{\lambda }}_{i}$ (i=1,⋯,L ) is an eigenvalue corresponding to the *i*th column vector $v_{i}^{L}$ of $V_{L}$ and $v_{i}^{R}$ of $V_{R}$. With the results of the eigenvalue problem in use, (34) can be decomposed by the eigenvectors as follows:

(42) $$\begin{array}{cc} B(n) & =V_{R}\bar{\Lambda }V_{L}^{T}+V_{R}{\bar{\Lambda }}^{2}V_{L}^{T}+\cdots +V_{R}{\bar{\Lambda }}^{2^{n-1}}V_{L}^{T}\\ \; & =V_{R}H\left(\bar{\Lambda },n\right)V_{L}^{T}, \end{array}$$

where

(43) $$\begin{array}{c} H\left(a,n\right):=\sum_{k=0}^{n-1}a^{2^{k}}=a+a^{2}+a^{4}+\cdots +a^{2^{n-1}}. \end{array}$$

Note that, when n→∞, $H\left(a,\infty \right)=a+a^{2}+a^{4}+\cdots$ is one of the types called a Lacunary function ([43]). In addition, (42) is rewritten as follows:

(44) $$\begin{array}{c} B\left(n\right)=\sum_{i=1}^{L}v_{i}^{L}H\left({\bar{\lambda }}_{i},n\right){\left(v_{i}^{R}\right)}^{T}. \end{array}$$

When $\left|x\right|\geq 1$, (43) diverges because *n* is increased. In other words, to prevent ${\langle x\rangle }_{f,n}$ from diverging, the eigenvalues of B must satisfy $\left|{\bar{\lambda }}_{i}\right|<1$ for i=1,⋯,L. Generally, (30) or (32) diverges because some eigenvalues with $\left|{\bar{\lambda }}_{i}\right|\geq 1$ exist. To control this divergence, we introduce the hyperparameter ϵ≥0 and modify (30) as follows:

(45) $$\begin{array}{c} {\langle x^{\epsilon }\rangle }_{f,n}=\mu +\sum_{p=0}^{n-1}e^{-\epsilon p}C_{p}f. \end{array}$$

When ϵ=0, (45) becomes (30); thus, ${\langle x^{0}\rangle }_{f,n}={\langle x\rangle }_{f,n}$. A well-chosen ϵ>0 can prevent (45) from diverging. In addition, we define the following:

(46) $$\begin{array}{cc} H^{\epsilon }\left(a,n\right) & :=\sum_{n=0}^{\infty }e^{-\epsilon n}a^{2^{n}}\\ \; & =a+e^{-\epsilon }a^{2}+e^{-2\epsilon }a^{4}+e^{-3\epsilon }a^{8}+\cdots . \end{array}$$

Thus, the modified *n*-LRM is expressed as follows:

(47) $$\begin{array}{cc} {\langle x^{\epsilon }\rangle }_{f,n} & :=\mu +C^{\epsilon }\left(n\right)f, \end{array}$$

(48) $$\begin{array}{cc} C^{\epsilon }\left(n\right) & :=\sum_{p=0}^{n-1}e^{-\epsilon p}C_{p}=\Sigma^{-1}B^{\epsilon }\left(n\right), \end{array}$$

(49) $$\begin{array}{cc} B^{\epsilon }\left(n\right) & :=\sum_{i=1}^{L}v_{i}^{L}H^{\epsilon }\left({\bar{\lambda }}_{i},n\right){\left(v_{i}^{R}\right)}^{T}. \end{array}$$

**Figure 2:**
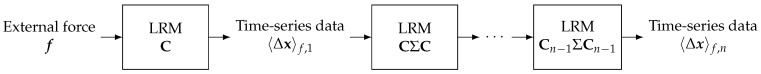
Data generation using the MLRM.

In this study, (47) is used to generate the data in the proposed MLRM. Figure 2 shows a schematic representation of the proposed MLRM. Here, the proposed MLRM is constructed by extending the LRM to multiple layers, which means that the MLRM incorporates the output data of the LRM iteratively.

## 3. Method

Section 3.1 describes the principal component analysis (PCA) used for estimating external forces, introducing a dimensionality reduction technique. Section 3.2 details the estimation of two time covariance matrices, which are used for PCA and for data generation in the proposed MLRM. Section 3.3 outlines the method used for estimating external force vectors. Section 3.4 presents the approach that uses the proposed MLRM for data generation. Section 3.5 introduces dynamic time warping (DTW), which is employed to evaluate the data generated by the proposed MLRM.

### 3.1. PCA of Two-Time Covariance Matrix

PCA is employed in Section 3.3 as part of the procedure for estimating external forces. In this section, we consider a real–symmetric eigenvalue problem of C with an orthonormal condition that is similar to those found in PCA.

(50) $$\begin{array}{cc} CV & =V\Lambda , \end{array}$$

(51) $$\begin{array}{cc} VV^{T} & =V^{T}V=I. \end{array}$$

Here, we obtain V, Λ, and I, which are the eigenvector, eigenvalue, and unit matrices, respectively. Note that Λ is a diagonal matrix whose *i*th diagonal element $\lambda_{i}$ (i=1,⋯,L ) is the variance of the *i*th PC, and the *i*th column vector $v_{i}$ of V is the corresponding eigenvector.

We consider approximating C using only a few top components with the contribution ratio of the eigenvalues. Typically, $\lambda_{i}$ is sorted in descending order such that the first PC shows the greatest variance $\lambda_{1}$, and the corresponding column vector $v_{1}$ of $V=(v_{1},\cdots v_{L})$ indicates the eigenvector of $\lambda_{1}$.

### 3.2. Estimation of Two-Time Covariance Matrix

Here, let $x^{d}:={\left(x_{1}^{d},x_{2}^{d},\cdots ,x_{N}^{d}\right)}^{T}\in R^{L}$ for d=1,2,⋯,D be the vector of *M*-dimensional stock price vectors in the intraday on the date indexed by *d*, where d=1 and d=D represent the start and end dates of the interval used in the estimation period, respectively. In addition, let *k* be a positive integer, and consider the interval starting from the *k* days prior to the current date, *d* through *d*. Then, the two-time covariance matrix $C^{d:k}$, estimated by the sample data from d−k+1 through *d*, is defined as follows:

(52) $$\begin{array}{cc} C^{d:k} & :=\frac{1}{k}\sum_{i=d-k+1}^{d}\Delta x^{i}{\left(\Delta x^{i}\right)}^{T}, \end{array}$$

(53) $$\begin{array}{cc} \Delta x^{i} & :=x^{i}-\mu^{d:k}, \end{array}$$

(54) $$\begin{array}{cc} \mu^{d:k} & :=\frac{1}{k}\sum_{i=d-k+1}^{d}x^{i}. \end{array}$$

### 3.3. Estimation of External Force Vector

Here, we consider $f^{d}$ and ${\langle \Delta x^{d}\rangle }_{f}$ for d=1,2,⋯,D to be the external force vector and the observed displacement vector in the intraday on day *d*, respectively. Using the observed ${\langle \Delta x^{d}\rangle }_{f}$ and the estimated $C^{d-1,k}$, we can estimate $f^{d}$ by solving the following system of linear equations using the LRM:

(55) $${\langle \Delta x^{d}\rangle }_{f}=C^{d-1:k}f^{d}.$$

However, the number L=N×M is large; thus, $C^{d-1:k}$ may generally fall in an ill-conditioned matrix. Therefore, it is difficult to solve (55) directly. To address this problem, we apply a dimension reduction technique to (55) via PCA (refer to Section 3.1). Let $\Lambda^{d-1}$ and $V^{d-1}$ be the eigenvalue and eigenvector matrix of $C^{d-1:k}$, respectively. In this case, we fix the parameter $\lambda_{c}^{d-1}$ to cut off the eigenvalue close to zero. Let $L_{c}^{d-1}$ be a number of elements in the set $\left\{\lambda_{i}^{d-1}|\lambda_{i}^{d-1}>\lambda_{c}^{d-1},i=1,\cdots ,L\right\}$, i.e., $\lambda_{1}^{d-1}>\cdots >\lambda_{L_{c}^{d-1}}^{d-1}>\lambda_{c}^{d-1}$. We consider the projection with ${\tilde{V}}^{d-1}:=\left(v_{1}^{d-1},\cdots ,v_{L_{c}^{d-1}}^{d-1}\right)$ as follows:

(56) $${\langle \Delta {\tilde{x}}^{d}\rangle }_{f}:={\left({\tilde{V}}^{d-1}\right)}^{T}{\langle \Delta x^{d}\rangle }_{f}\in R^{L_{c}^{d}}.$$

Then, (55) for this projection space is expressed as follows:

(57) $${\langle \Delta {\tilde{x}}^{d}\rangle }_{f}={\tilde{C}}^{d-1:k}{\tilde{f}}^{d},$$

where

(58) $$\begin{array}{cc} {\tilde{C}}^{d-1:k} & :={\left({\tilde{V}}^{d-1}\right)}^{T}C^{d-1:k}{\tilde{V}}^{d-1}\in R^{L_{c}^{d}\times L_{c}^{d}}, \end{array}$$

(59) $$\begin{array}{cc} {\tilde{f}}^{d} & :={\left({\tilde{V}}^{d-1}\right)}^{T}f^{d}\in R^{L_{c}^{d}}. \end{array}$$

By setting $\lambda_{c}^{d-1}$ successfully and removing the components with small eigenvalues, ${\tilde{C}}_{c}^{d-1:k}$ is well conditioned.

Finally, to convert ${\tilde{f}}^{d}$ to $f^{d}$ approximately in the original dimension *L*, the following equation is applied for d=1,⋯,D:

(60) $$f^{d}\approx {\tilde{V}}^{d}{\tilde{f}}^{d}\in R^{L}.$$

We then use the force vectors estimated using (60). In addition, we estimate $\Sigma =\langle ff^{T}\rangle$ as follows:

(61) $$\begin{array}{cc} \Sigma & :=\frac{1}{D}\sum_{d=1}^{D}f^{d}{\left(f^{d}\right)}^{T}\in R^{L\times L}. \end{array}$$

### 3.4. Data Generation by MLRM

Here, we explain the data generation process using the proposed MLRM. Let d∈{1,⋯,D} be a fixed day, and let $f^{d}$ for d=1,⋯,D be the approximated external force vectors given by (60). Then, we obtain the following data generation equation using the MLRM:

(62) $$\begin{array}{cc} {\langle x^{\epsilon ,d}\rangle }_{f,n} & :=\mu^{d:k}+C^{\epsilon ,d:k}\left(n\right)f^{d}, \end{array}$$

(63) $$\begin{array}{cc} C^{\epsilon ,d:k}\left(n\right) & :=\sum_{p=0}^{n-1}e^{-\epsilon p}C_{p}^{d:k}, \end{array}$$

where

(64) $$\begin{array}{cc} C_{0}^{d:k} & :=C^{d:k}, \end{array}$$

(65) $$\begin{array}{cc} C_{p}^{d:k} & :=C_{p-1}^{d:k}\Sigma C_{p-1}^{d:k}, \end{array}$$

for p=1,⋯,n−1, and ϵ≥0 is selected to prevent (62) from diverging. Note that $C^{d:k}$ and Σ are calculated using (52) and (61), respectively.

### 3.5. DTW

In this study, we use DTW [44] to compare the generated and real time-series data. DTW represents the distance between the generated time-series data $x:={\left\{x_{1},\cdots ,x_{L}\right\}}^{T}\in R^{L}$ and the real time-series data $y:={\left\{y_{1},\cdots ,y_{L}\right\}}^{T}\in R^{L}$. Note that a small DTW value means that the dynamics of the two sets of time-series data are similar. In addition, the cost function associated with a warping path $p:=(\left(p^{1},p^{1}\right),\cdots ,\left(p^{L},p^{L}\right))$ is obtained by computing with respect to all pairwise distances $\left\{d\left(x_{i},y_{j}\right)|i,j=1,\cdots ,L\right\}\in R^{L\times L}$:

(66) $$\begin{array}{c} c_{p}\left(x,y\right):=\sum_{i=1}^{L}c\left(X_{p^{i}},Y_{p^{i}}\right). \end{array}$$

The warping path p∗ that incurs the minimum cost associated with the alignment is referred to as the optimal warping path. DTW is defined as the cost function with the optimal warping path as follows:

(67) $$\begin{array}{c} DTW\left(x,y\right):=c_{p*}\left(x,y\right):=\min_{p}c_{p}\left(x,y\right). \end{array}$$

## 4. Experimental Results

### 4.1. Experimental Data

**Figure 3:**
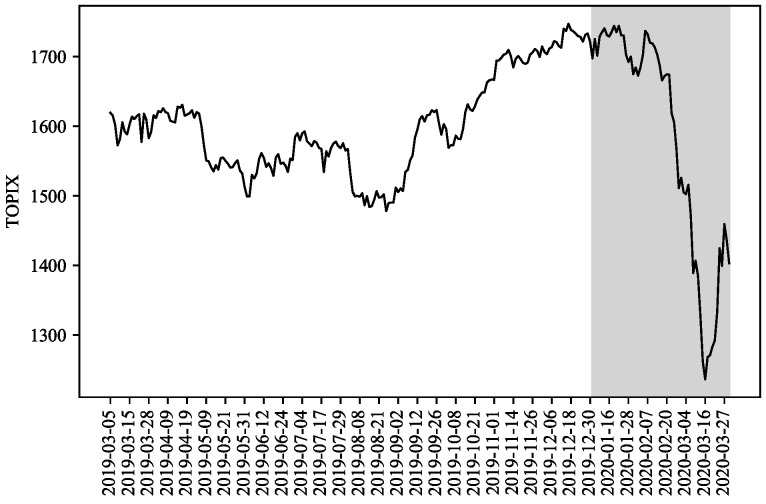
TOPIX: the black line represents the time evolution of the TOPIX from March 2019 to March 2020, while the gray region denotes the out-of-sample period (January 2020 to March 2020).

**Figure 4:**
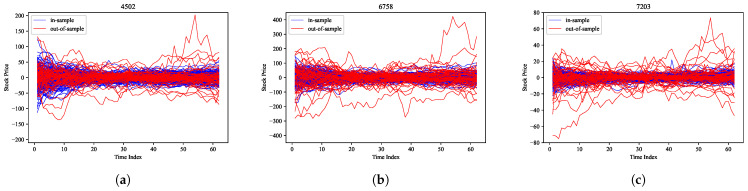
Time-series data of stock prices: (**a**) Takeda Pharmaceutical Co., Ltd. (4502), (**b**) Sony Corporation (6758), and (**c**) Toyota Motor Corporation (7203). The red line represents the in-sample data; the blue line denotes the out-of-sample data.

To evaluate the proposed model, we selected the Tokyo Stock Exchange (TSE) as the target market and used time-series data for stock prices extracted from the FLEX Full data which is a dataset that Japan Exchange Group (JPX) constructs with the real-time stock market data from the TSE on a daily basis and provides it as historical information. The Tokyo Stock Price Index (TOPIX) is a capitalization-weighted index of all companies listed in the First Section of the TSE. The black line in Figure 3 shows the time evolution in the TOPIX from March 2019 to March 2020. The COVID-19 shock caused the TOPIX to decline sharply in both February 2020 and March 2020. Here, we divided this period into two smaller periods without and with the COVID-19 shock, respectively, and we refer to these periods as the in-sample and out-of-sample periods, respectively. The latter period is shown as a gray region in Figure 3. As target stocks, we selected M=3 stocks, i.e., Takeda Pharmaceutical Co., Ltd. (4502), Sony Corporation (6758), and Toyota Motor Corporation (7203) from the group of stocks listed on the TSE between March 2019 and March 2020 and included in the TOPIX CORE 30, which is the stock price index comprising 30 stocks with considerably high market capitalization and liquidity among all stocks on the TSE. In addition, we selected 09:00 to 11:30 (morning session) and 12:30 to 15:00 (afternoon session) as the time zones and used snapshots of the stock price data every 5 min. This resulted in time-series data for a total of 62 time points (9:00 to 11:30 ( representing 31-time points) and 12:30 to 15:00 (representing 31-time points) per day. In addition, all stock prices are preprocessed to make their averages zero in the time direction, which are denoted $x_{t,m}$ for t=1,⋯,62 and m=1,⋯,3. Thus, $\sum_{t=1}^{62}x_{t}^{d}=0\in R^{3}$ and the dimension of $x^{d}$ for d=1,⋯,D was 3×62=186, and the index of this dimension was referred to as a Time-Stock Index. The red and blue lines in Figure 4 show the time-series data for the three stock prices in the in-sample and out-of-sample periods, respectively.

### 4.2. Estimation of External Force Vectors

In this evaluation, we set D=731 and k=200, and we estimated ${\left\{C^{d-1:k}\right\}}_{d=1,\cdots D}$ using (52). In addition, we determined the number *D* of the eigenvalues ${\left\{L_{c}^{d-1}\right\}}_{d=1,\cdots ,D}$, such that the sum of eigenvalue ratios for ${\left\{C^{d-1:k}\right\}}_{d=1,\cdots D}$ just exceeds 0.95. This value of *D* was employed to remove eigenvalues that are close to zero. Using the number of reduced eigenvalues ${\left\{L_{c}^{d}\right\}}_{d=1,\cdots ,D}$, we calculated the reduced matrix ${\left\{{\tilde{C}}^{d-1:k}\right\}}_{d=1,\cdots ,D}$ with the projection vectors ${\left\{{\tilde{V}}^{d-1:k}\right\}}_{d=1,\cdots ,D}$ (refer to (58)).

The set ${\left\{{\langle x^{d}\rangle }_{f}\right\}}_{d=1,\cdots ,D}$ is the real stock price data, which we considered to be under the influence of external forces. We calculated ${\left\{{\langle \Delta x^{d}\rangle }_{f}\right\}}_{d=1,\cdots ,D}$ by subtracting the expected values of ${\left\{{\langle x^{d}\rangle }_{f}\right\}}_{d=1,\cdots ,D}$. We then obtained ${\left\{{\langle \Delta {\tilde{x}}^{d}\rangle }_{f}\right\}}_{d=1,\cdots ,D}$ using the projection vectors ${\left\{{\tilde{V}}^{d-1:k}\right\}}_{d=1,\cdots ,D}$ (refer to (56)). Next, we estimated ${\left\{{\tilde{f}}^{d}\right\}}_{d=1,\cdots ,D}$ using (57).

**Figure 5:**
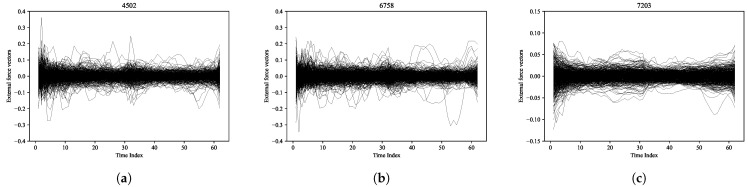
External force vectors: (**a**) Takeda Pharmaceutical Co., Ltd. (4502), (**b**) Sony Corporation (6758), and (**c**) Toyota Motor Corporation (7203) for 1,⋯,D.

**Figure 6:**
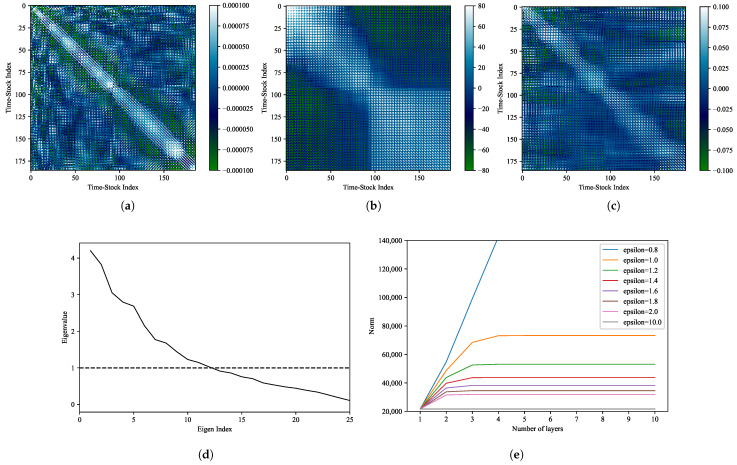
Matrices using the MLRM and its convergence: (**a**) $\Sigma =\langle ff^{T}\rangle$, (**b**) $C^{d:k}$, (**c**) $B^{d:k}$, (**d**) eigenvalues of $B^{d:k}$ and (**e**) norms of $C^{d:k}$ (d=D and k=200).

Note that ${\left\{{\tilde{f}}^{d}\right\}}_{d=1,\cdots ,D}$ are vectors in a lower-dimensional space; thus, we approximated ${\left\{f^{d}\right\}}_{d=1,\cdots ,D}$ by transforming them back to the original dimension using (60). From these transformations, we obtained various patterns of external forces based on the historical data. The results for Takeda Pharmaceutical Co., Ltd. (Tokyo, Japan) (4502), Sony Corporation (Tokyo, Japan) (6758), and Toyota Motor Corporation (Toyota, Japan) (7203) are shown in Figure 5a–c, respectively. Using the calculated external forces, we computed Σ using (61), and the results are presented in Figure 6a. As can be seen, the matrix Σ is symmetric, and it exhibits a strong positive correlation near the diagonal.

### 4.3. Estimation of C and B

We selected the day d=D, i.e., 30 December 2019, which is the last date in the in-sample period. The corresponding two-time covariance matrix $C^{d:k}$ is shown in Figure 6b, which shows the existence of high values near the diagonal and a negative correlation between the morning and afternoon sessions.

Figure 6c shows the matrix $B=\Sigma C^{d:k}$ estimated by (35), which is an asymmetric matrix. Here, we obtained the eigenvalues by solving the eigenvalue problem for B using (39) to (41). Figure 6d shows the eigenvalues of B from 1 to 25. As can be seen, the eigenvalues of B include values that are greater than 1.0, which means that the MLRM diverges as the number of layers increases. To address this divergence problem, data generation using the MLRM was performed by introducing the parameter ϵ to realize the sufficient suppression of divergence.

Next, we examined the convergence speed, depending on ϵ, using $C^{\epsilon ,d:k}\left(n\right)$ in (63). Here, we evaluated how the norm of $C^{\epsilon ,d:k}\left(n\right)$ changed as we increased the number of layers, with ϵ values ranging from 0.8 to 100.0. Figure 6e shows the results of the norm values of $C^{\epsilon ,d:k}\left(n\right)$. The results confirm that, as the number of layers increases, a large ϵ value typically approaches the SLRM, and a small ϵ value diverges. In this study, we selected ϵ=1 among candidates for which the convergence of the $C^{\epsilon ,d:k}\left(n\right)$ norm was confirmed. In addition, we confirmed that 10 layers are sufficient for convergence; thus, the number of layers for the MLRM was set to n=10, thereby resulting in a 10-layer LRM (10-LRM).

### 4.4. *Data Generation Using MLRM*

**Figure 7:**
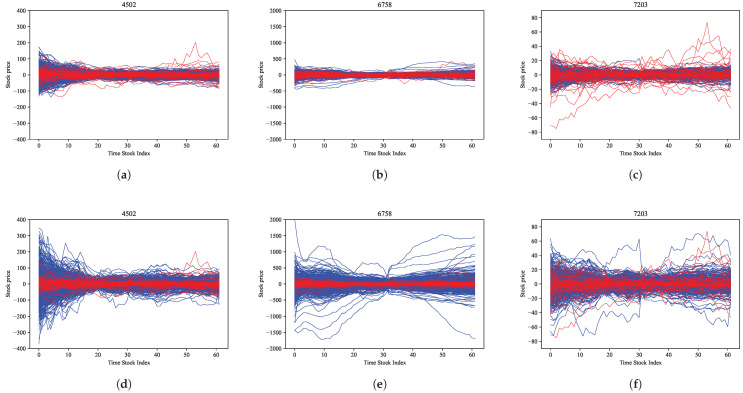
Comparison of the stock price data generated via the SLRM and the MLRM. (**a**–**c**) show the results of the SLRM, and (**d**–**f**) show the results of the MLRM for three representative stocks: (**a**,**d**) Takeda Pharmaceutical Co., Ltd. (4502); (**b**,**e**) Sony Corporation (6758); and (**c**,**f**) Toyota Motor Corporation (7203). The blue lines represent the generated stock price data via the SLRM and MLRM (10-layer MLRM), and the red lines represent the real out-of-sample data.

In this evaluation, we set ϵ=1.0 and n=10, and the data generation for each stock by the proposed MLRM was performed using (62). Figure 7 shows the data generation results for Takeda Pharmaceutical Co., Ltd., Sony Corporation, and Toyota Motor Corporation, respectively. In Figure 7, the upper (a,b,c) and lower (d,e,f) graphs show the size of the data generated via the SLRM and MLRM (10-LRM) (blue lines), respectively, and the red lines represent the real out-of-sample data, which include samples with very large fluctuations due to the COVID-19 pandemic. A comparison of the SLRM and MLRM methods shows that the MLRM generates time-series with larger fluctuations than the SLRM. The time-series data generated via the proposed MLRM indicate that the out-of-sample fluctuations are covered. These results suggest that the data generation results obtained using the proposed MLRM contain information about the dynamics that is similar to that of the out-of-sample data.

**Figure 8:**
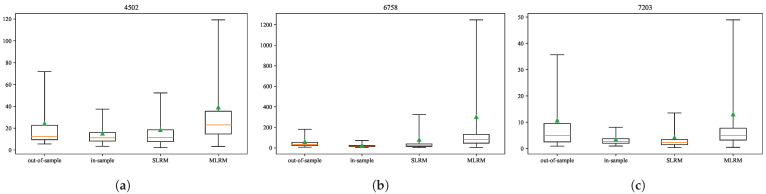
Box plots of the percentiles of the stock price’s SD: (**a**) Takeda Pharmaceutical Co., Ltd. (4502), (**b**) Sony Corporation (6758), and (**c**) Toyota Motor Corporation (7203). The black horizontal lines represent the 0th, 25th, 75th, and 100th percentiles (from the bottom), and the orange line indicates the 50th percentile. The triangles represent the averages.

Figure 8 shows box plots for the percentile of the stock price’s standard deviation (SD) calculated using the in-sample data, out-of-sample data, SLRM, and proposed MLRM. As can be seen, the fluctuations in the out-of-sample data are much larger than those in the in-sample data, which only partially capture the fluctuations observed in the out-of-sample period. The data generated via the SLRM show an improvement compared to the in-sample data, except for Sony Corporation (6752); however, the fluctuations captured via the SLRM do not fully account for those observed in the out-of-sample data. In contrast, the proposed MLRM generated significantly larger fluctuations, thereby effectively covering the out-of-sample fluctuations. These results demonstrate that the data generated via the proposed MLRM are more similar to the out-of-sample data than to the other sample data.

### 4.5. Comparison of DTW

**Figure 9:**
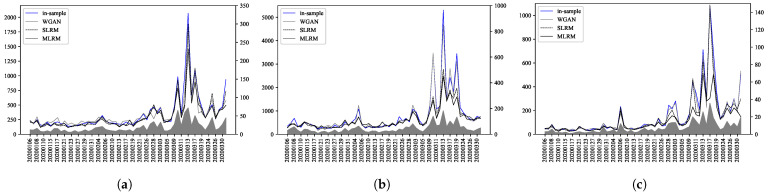
Comparison of daily minDTW for (**a**) Takeda Pharmaceutical Co., Ltd. (4502), (**b**) Sony Corporation (6758), (**c**) Toyota Motor Corporation (7203). The minDTW (left axis) is shown for the in-sample (blue line), SLRM (black dotted line), proposed MLRM (black line), and WGAN (gray line). The light gray areas represent the SD of the stock prices (right axis).

We employed DTW to evaluate the similarity between the out-of-sample stock price data and the generated time-series dynamics. We also compared the results with those obtained using the WGAN. The WGAN is a machine learning method that generates time-series data by learning the in-sample data distribution through adversarial training. These comparisons are illustrated in Figure 9. We define minDTW as the minimum DTW distance between the out-of-sample data and any of the generated data samples. The left axis of Figure 9 shows the minDTW value for each day.

Here, a smaller minDTW indicates higher similarity between the two time-series data. The right axis shows the SD of the stock price. Notably, there are areas that exhibit high SD, particularly between 10 March 2020, and 19 March 2020, which is likely due to market disruptions caused by the COVID-19 pandemic. The in-sample results exhibit very high minDTW during periods heavily influenced by COVID-19, which indicates that the in-sample data does not contain time-series data that are similar to those for the COVID-19 period, thereby contributing to the high minDTW. The SLRM obtained better results than the in-sample results because the extrapolation representation of the SLRM, which is a first-order approximation relative to external forces, works very effectively. However, the proposed MLRM realized the most significant improvement in the minDTW during the COVID-19 period, which suggests that the MLRM captured large fluctuation dynamics effectively. In contrast, the results obtained via the WGAN were similar to the in-sample results, with higher minDTW during the COVID-19 period. These findings indicate that it is difficult to generate time-series data that are similar to those during the COVID-19 period using machine learning models that are dependent on historical data.

Table 1, Table 2 and Table 3 show the minDTW for the top 10 highest-SD days for each stock in the out-of-sample data, sorted by the SD, where the bold numbers indicate the lowest values in each column. The results in these tables confirm that the MLRM is able to generate data closer to the out-of-sample data than other methods on high-SD days. As expected, the MLRM successfully generates data with large fluctuations.

Next, Table 4, Table 5 and Table 6 show the minDTW for the top 10 lowest-SD days for each stock in the out-of-sample data, also sorted by the SD. The results confirm that the SLRM is able to generate data closer to the out-of-sample data than other methods on low-SD days. This suggests that the SLRM is effective for generating data without large fluctuations.

In summary, the evaluation results demonstrate that the proposed MLRM is a more effective tool for capturing the dynamics of anomalous events, such as those we faced during the COVID-19 pandemic. Moreover, by utilizing data generated from both the SLRM and MLRM, we can obtain artificial data that reflects the dynamics of both small and large fluctuations.

### 4.6. Comparison of Real Dynamics and Data Generated by MLRM

**Figure 10:**
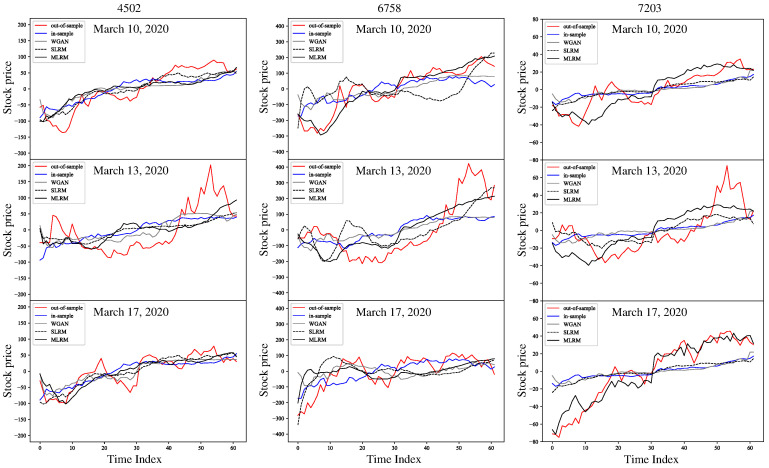
Comparison of dynamics on 10 March 2020, 13 March 2020, and 17 March 2020. The time-series data for the out-of-sample (red line), in-sample (blue line), WGAN (grey line), SLRM (dotted black line), and MLRM (black line) are shown. Each was selected based on the minDTW.

Furthermore, we compared the stock price dynamics of the generated and real data. Here, we focused on 10 March, 13 March, and 17 March 2020, when stock prices experienced significant fluctuations due to the impact of the COVID-19 pandemic. Figure 10 compares the dynamics between the real and generated data for the in-sample, WGAN, SLRM, and proposed MLRM, where each was selected based on the minDTW.

On 10 March, 13 March, and 17 March 2020, the minDTW values of the MLRM were lower than those of the in-sample, WGAN, and SLRM in most cases from Table 1, Table 2 and Table 3. Specifically, as shown in Figure 10, the stock price dynamics for Sony Corporation (6758) on 10 March 2020, and Toyota Motor Corporation (7203) on 17 March 2020, generated via the proposed MLRM were highly similar to the real data. This indicates that the MLRM faces challenges in generating stock price dynamics under highly uncertain conditions. Therefore, we expect that the MLRM will be applicable to various problems in the financial domain, e.g., risk management and scenario testing.

### 4.7. Tail Behavior of Log-Return Distributions

**Figure 11:**
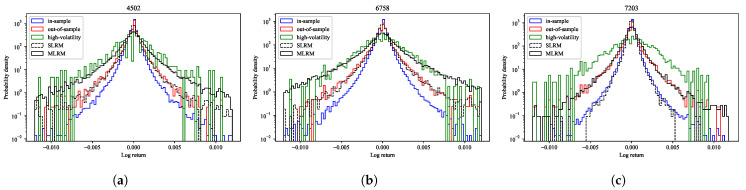
Distributions of log returns for three representative stocks: (**a**) 4502, (**b**) 6758, and (**c**) 7203. The vertical axis shows the probability density on a logarithmic scale; the horizontal axis indicates the log return. The empirical distributions are shown for the in-sample period (blue solid line), out-of-sample period (red solid line), and high-volatility period (green solid line). The distributions generated via the SLRM (black dotted line) and the MLRM (black solid line) are also displayed. The MLRM-generated distributions exhibit heavier tails and closely match the empirical distributions observed during high-volatility periods.

Figure 11 presents the distributions of log returns for three representative stocks (4502, 6758, and 7203). The vertical axis denotes the probability density on a logarithmic scale, while the horizontal axis corresponds to the log return. The empirical distributions for the in-sample period, out-of-sample period, and a subset of high-volatility observations are compared with those generated by the SLRM and the MLRM. The realized volatility used in this analysis is computed on a daily basis from intraday log returns sampled at 5 min intervals. Specifically, for each trading day, *d*, the realized volatility, $RV_{d}$, is defined as follows:

(68) $$RV_{d}=\sum_{t=1}^{T}r_{d,t}^{2},$$

where $r_{d,t}$ denotes the *t*-th 5-min log return on day *d*, and *T* is the number of such intervals within a trading day. The high-volatility sample is constructed by selecting out-of-sample observations for which the realized volatility exceeds the upper bound of the interquartile range (IQR), thereby isolating periods characterized by elevated market volatility. It is important to note that the SLRM and MLRM generate stock price series as zero-mean intraday fluctuations, lacking absolute price level information. To ensure consistency with the empirical data, we restore the price level by adding the mean of the in-sample stock price series to the generated outputs before computing the log returns. This procedure enables a meaningful comparison of return distributions across empirical and model-generated data. The distribution generated via the MLRM exhibits heavier tails than that produced using the SLRM, with tail behavior that lies between the empirical distributions of the out-of-sample period and the high-volatility sample. This finding indicates that the MLRM is capable of more effectively capturing the non-stationary dynamics and intricate correlation structures that emerge in the market under high-volatility conditions. By contrast, the SLRM yields a distribution with comparatively thinner tails, consistent with return behavior observed during more typical market regimes.

Table 7 shows the estimated excess kurtosis (κ) of the log-return distributions for stocks 4502, 6758, and 7203. The excess kurtosis κ of a random variable, *X*, with mean μ and standard deviation σ, is defined as

(69) $$\kappa :=\frac{E\left[{\left(X-\mu \right)}^{4}\right]}{\sigma^{4}}-3.$$

Excess kurtosis characterizes the heaviness of the distribution tails relative to a normal distribution. A normal distribution has κ=0; positive values of κ indicate heavier tails and a higher likelihood of extreme returns, while negative values indicate lighter tails. In the MLRM-generated data, increasing the number of model layers leads to higher kurtosis, enabling the model to capture extreme market fluctuations and the heavy-tailed behavior observed in real financial markets effectively.

The parameter γ, estimated from the log-return distributions and reported in Table 7, corresponds to the exponent of the power-law tail, characterizing the rate of decay in the distribution’s tails. Empirical studies of financial markets have shown that the tail behavior of log returns typically follows $P\left(|r|>x\right)∼x^{-\gamma }$, where the so-called cubic law suggests that γ is approximately 3 under ordinary market conditions—specifically in developed markets, characterized by high liquidity and institutional maturity. Values of γ lower than 3 indicate heavier tails, implying a higher frequency of extreme returns. In this study, we estimated the tail index using the Hill estimator with a truncation level of 7.5%, following the approach recommended in the literature for developed markets [36]. Our results show that γ is consistently below 3 across all stocks in both the out-of-sample data and the data generated via the MLRM, demonstrating that the model effectively captures the heavy-tailed nature observed in real financial markets.

**Figure 12:**
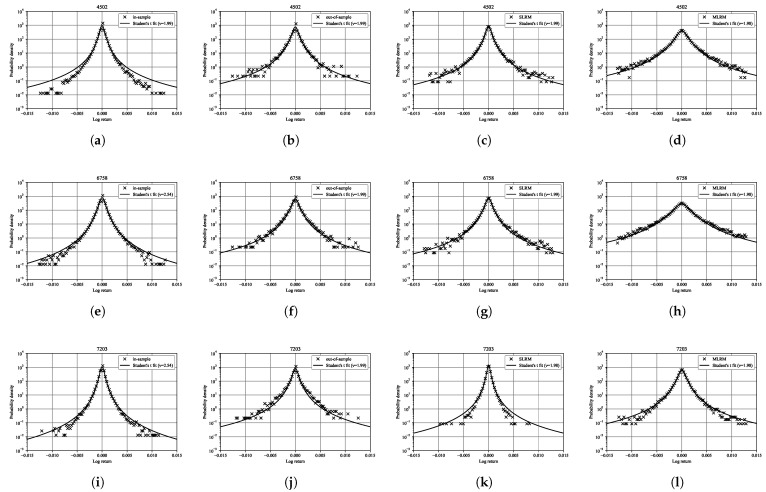
Student’s *t* distribution fits for the log-return distributions of three representative stocks (4502, 6758, and 7203). For each stock, four types of data are shown: in-sample data (**a**,**e**,**i**), out-of-sample data (**b**,**f**,**j**), data generated via the SLRM (**c**,**g**,**k**), and data generated via the MLRM (**d**,**h**,**l**). Each distribution is fitted with a Student’s *t* distribution, and the estimated degrees of freedom ν are indicated in the legend. The probability density is shown on a logarithmic scale to highlight the heavy-tailed behavior. The results demonstrate that both the SLRM and the MLRM can reproduce the heavy-tailed nature of real financial returns, with the MLRM providing a better fit across all three stocks.

Given that the normal distribution cannot adequately capture such heavy-tailed behavior, we follow the approach of Praetz [41], who demonstrated that heavy-tailed financial returns are well described by Student’s *t* (or *q*-Gaussian) distribution. Figure 12 presents the results of fitting Student’s *t* distributions to the log-return distributions of three representative stocks (4502, 6758, and 7203). The *Student’s t distribution* with ν degrees of freedom is defined by the probability density function

(70) $$p\left(x|\nu \right):=\frac{\Gamma \left(\frac{\nu +1}{2}\right)}{\sqrt{\nu \pi }\;\Gamma \left(\frac{\nu }{2}\right)}{\left(1+\frac{x^{2}}{\nu }\right)}^{-\frac{\nu +1}{2}},$$

where Γ(·) denotes the gamma function. This distribution is symmetric and bell-shaped like the normal distribution but exhibits heavier tails, particularly for small values of ν. The degree of freedom, ν, governs the tail heaviness: smaller values of ν indicate heavier tails and a higher frequency of extreme returns. The fits are shown for the in-sample period, the out-of-sample period, and for data generated via the SLRM and the MLRM, with the estimated degrees of freedom ν indicated in each panel. The results show that the estimated degrees of freedom ν remain consistently around 2 across all data sets, reflecting the presence of heavy-tailed behavior in both empirical and model-generated distributions. Both the SLRM and the MLRM successfully reproduce this characteristic, with the MLRM providing slightly better alignment with the empirical tail behavior in some cases. These findings suggest that the models effectively capture the high frequency of extreme returns observed in real financial markets.

### 4.8. Tail Behavior of Realized Volatility Distributions

**Figure 13:**
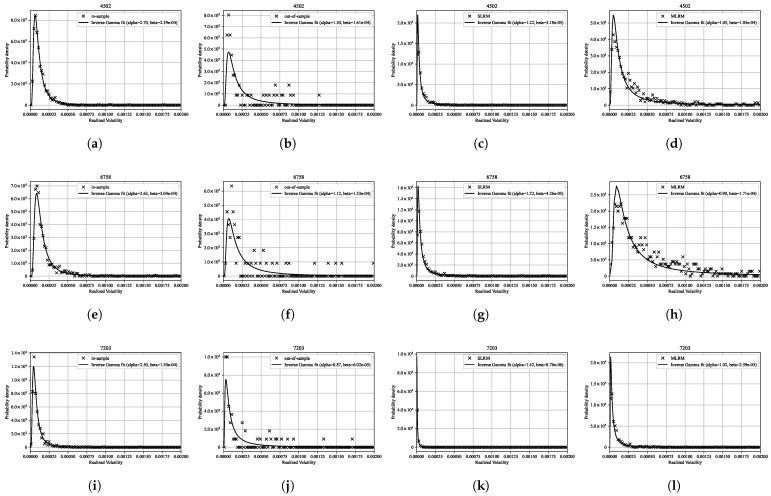
Distributions of realized volatility for three representative stocks (4502, 6758, and 7203), along with their corresponding inverse gamma distribution fits. For each stock, results are presented for the in-sample period (**a**,**e**,**i**), the out-of-sample period (**b**,**f**,**j**), and for data generated via the SLRM (**c**,**g**,**k**) and the MLRM (**d**,**h**,**l**). The estimated parameters of the inverse gamma distribution (α, β) are reported in each panel. These results demonstrate that, while the SLRM tends to underestimate the probability density in the tails, the MLRM more accurately captures the heavy-tailed characteristics of the empirical volatility distributions observed in real financial markets.

Figure 13 presents the distributions of realized volatility for three representative stocks (4502, 6758, and 7203), along with their corresponding inverse gamma distribution fits. The inverse gamma distribution, commonly used to model heavy-tailed positive variables such as volatility, is defined by its probability density function:

(71) $$p\left(x|\alpha ,\beta \right):=\frac{\beta^{\alpha }}{\Gamma (\alpha )}x^{-\alpha -1}\exp\left(-\frac{\beta }{x}\right),\;x>0,$$

where α is the shape parameter and β is the scale parameter. For each stock, the distributions are shown for the in-sample period, out-of-sample period, and for data generated via the SLRM and the MLRM. The MLRM-generated distributions closely match the empirical distributions, particularly in the tails, across all three stocks. It should be noted that the out-of-sample distributions exhibit greater variability due to the limited number of data points available, which results in less stable fits to the inverse gamma distribution.

Furthermore, it is known that there exists a theoretical relationship between the power-law exponent α of the inverse gamma distribution fitted to the realized volatility and the degrees of freedom ν of the Student’s t distribution fitted to the log-return distribution, given by ν=2α [45]. This relationship arises from models that describe the log returns as a mixture distribution driven by stochastic volatility, implying consistency between the tail behaviors of returns and volatility. In our results, the estimated ratio ν/α is generally close to 2 (see Table 7), which is in good agreement with the theoretical expectation. This observation further supports the conclusion that the data generated via the MLRM successfully capture both the tail behavior of returns and their relationship to the underlying volatility structure observed in real financial markets.

## 5. Conclusions

This paper has proposed the MLRM to generate time-series data. The proposed MLRM focuses on generating data for extreme fluctuations and anomalous dynamics and is constructed by extending the conventional SLRM to multiple layers. In this study, we evaluated the effectiveness of the proposed model by generating stock price data for the COVID-19 period using pre-COVID-19 pandemic data. The results demonstrated that the stock price data generated via the proposed MLRM exhibited a higher degree of similarity to real data during the COVID-19 period compared to other methods. However, because the MLRM estimates external forces based on historical data, it may be challenging to apply the MLRM to phenomena, such as regime switching, where the external forces driving future stock prices differ substantially from those observed historically. Nonetheless, this limitation may be overcome in future research by devising methodologies that model external forces independently of historical data.

In addition to generating realistic price trajectories, we further evaluated the tail behavior of the log-return and realized volatility distributions. The MLRM-generated data exhibited heavier tails in the log-return distributions, closely matching the empirical behavior observed during COVID-19 periods. The estimated power-law exponent γ and excess kurtosis κ confirm that the MLRM successfully reproduces the heavy-tailed nature of financial returns, which is critical for modeling extreme market movements. Moreover, the degrees of freedom ν of the Student’s *t* distribution fitted to the generated returns remained consistently low (around 2), which is consistent with empirical data, further supporting the model’s ability to replicate extreme return behavior.

Similarly, for realized volatility, the MLRM accurately captured the heavy-tailed nature of the empirical distributions, as evidenced by the fit to the inverse gamma distribution. Importantly, the theoretical relationship ν=2α between the volatility and return tail parameters was approximately satisfied by the MLRM-generated data, suggesting that the model can coherently capture the joint dynamics of returns and volatility observed in real markets.

Although it is generally difficult for machine learning models to generate data that is not included in the historical dataset, the MLRM can pre-generate rare events with extreme fluctuations, such as the COVID-19 pandemic. We believe that the artificial data reflecting both small and large fluctuations generated by the SLRM and the MLRM can be applied to various domains. In particular, the ability of the MLRM to reproduce realistic tail behavior is especially valuable for applications in financial risk management, stress testing, and scenario generation involving extreme market events. The results of this study have several practical implications for financial modeling and risk management. The proposed MLRM provides a transparent and computationally efficient framework for generating realistic synthetic financial time-series data, which can be utilized for scenario analysis, stress testing, and the development of algorithmic trading strategies. Furthermore, because the MLRM is based on explicit causal relationships, it offers enhanced interpretability relative to black-box machine learning models, making it particularly suitable for applications where model transparency is required, such as regulatory compliance and model validation. The ability to generate data that replicates extreme market fluctuations also enables more robust evaluation of risk measures and financial products under rare but critical market conditions. We believe that further development of the MLRM and related methodologies will contribute to more robust and interpretable approaches for modeling extreme events in financial markets.

## Figures and Tables

**Table 1:** minDTW of the top 10 highest-SD days for 4502. Bold indicates the minimum value among the methods in each row.

No	Date	SD	In-Sample	SLRM	MLRM	WGAN
1	13 Mar 2020	71.8	2070.3	1882.6	**1457.9**	1756.3
2	10 Mar 2020	65.3	983.3	930.0	**793.2**	914.1
3	12 Mar 2020	58.5	1014.0	505.3	**472.4**	724.2
4	17 Mar 2020	50.7	1095.3	1131.1	**892.4**	1032.5
5	25 Mar 2020	45.5	506.8	698.6	**488.3**	597.7
6	31 Mar 2020	44.7	936.2	742.0	**495.3**	577.2
7	28 Feb 2020	34.4	461.9	452.3	504.1	**265.2**
8	3 Mar 2020	33.0	454.1	**366.9**	411.1	465.6
9	18 Mar 2020	29.5	657.4	641.3	505.0	**362.0**
10	27 Feb 2020	28.1	426.1	407.4	**357.1**	373.7

**Table 2:** minDTW of the top 10 highest-SD days for 6752. Bold indicates the minimum value among the methods in each row.

No	Date	SD	In-Sample	SLRM	MLRM	WGAN
1	13 Mar 2020	182.1	5306.8	2778.1	**2479.9**	4672.5
2	10 Mar 2020	140.1	3463.7	1610.9	**1440.9**	3468.5
3	19 Mar 2020	134.0	3449.5	1972.2	**1596.6**	3109.9
4	17 Mar 2020	102.2	2425.0	**1776.4**	1894.9	2808.9
5	2 Mar 2020	81.0	1075.0	1009.3	**954.2**	1243.7
6	9 Mar 2020	74.6	1134.3	**928.8**	935.2	1229.9
7	12 Mar 2020	71.6	1197.0	1251.1	**1071.2**	1242.7
8	18 Mar 2020	70.4	1823.5	1538.7	**1249.5**	1647.9
9	11 Mar 2020	66.9	1084.9	680.6	**679.3**	892.4
10	5 Feb 2020	63.5	1076.0	748.6	**722.5**	1235.7

**Table 3:** minDTW of the top 10 highest-SD days for 7203. Bold indicates the minimum value among the methods in each row.

No	Date	SD	In-Sample	SLRM	MLRM	WGAN
1	17 Mar 2020	35.7	1084.8	1086.8	**302.4**	1049.7
2	13 Mar 2020	25.8	711.7	610.7	**511.5**	668.8
3	10 Mar 2020	19.9	438.1	455.1	**230.2**	471.0
4	31 Mar 2020	19.6	506.9	298.7	**147.4**	531.6
5	18 Mar 2020	18.8	703.0	519.9	**501.2**	611.0
6	11 Mar 2020	14.9	343.9	179.6	**163.8**	299.5
7	25 Mar 2020	14.7	261.0	253.2	**220.5**	275.8
8	3 Mar 2020	13.5	279.9	**132.2**	142.0	260.7
9	2 Mar 2020	13.5	213.4	203.4	**156.1**	225.9
10	27 Mar 2020	12.7	298.8	257.9	**222.0**	294.6

**Table 4:** minDTW of the top 10 lowest-SD days for 4502. Bold indicates the minimum value among the methods in each row.

No	Date	SD	In-Sample	SLRM	MLRM	WGAN
1	27 Jan 2020	5.6	158.8	**144.9**	145.3	174.6
2	22 Jan 2020	5.8	137.9	123.2	**113.5**	166.1
3	23 Jan 2020	5.8	141.0	**128.2**	136.9	192.4
4	15 Jan 2020	6.4	145.9	137.7	**135.9**	198.1
5	10 Jan 2020	6.8	150.6	**137.8**	164.2	174.9
6	19 Feb 2020	6.9	166.7	147.2	**134.0**	180.4
7	20 Jan 2020	7.1	182.2	**144.1**	163.2	147.0
8	9 Jan 2020	7.7	159.5	121.8	**115.8**	134.7
9	28 Jan 2020	7.7	176.5	**160.0**	178.2	228.2
10	30 Jan 2020	7.8	206.9	**177.1**	209.0	226.0

**Table 5:** minDTW of the top 10 lowest-SD days for 6552. Bold indicates the minimum value among the methods in each row.

No	Date	SD	In-Sample	SLRM	MLRM	WGAN
1	20 Jan 2020	7.1	222.7	**172.7**	247.1	308.4
2	23 Jan 2020	11.0	**255.2**	269.8	319.3	387.7
3	14 Feb 2020	12.0	312.5	**285.1**	337.3	383.3
4	28 Jan 2020	12.7	301.5	**288.1**	301.6	363.5
5	10 Jan 2020	15.8	**301.6**	344.6	382.6	404.3
6	10 Feb 2020	16.2	336.5	**322.4**	459.9	411.5
7	24 Jan 2020	19.1	**258.2**	268.5	275.7	321.2
8	21 Jan 2020	19.2	293.1	**263.1**	366.8	317.4
9	5 Mar 2020	19.6	400.1	**358.3**	405.1	383.9
10	21 Feb 2020	19.7	**359.5**	382.5	387.9	377.7

**Table 6:** minDTW of the top 10 lowest-SD days for 7203. Bold indicates the minimum value among the methods in each row.

No	Date	SD	In-Sample	SLRM	MLRM	WGAN
1	16 Jan 2020	0.9	33.8	**24.4**	29.6	34.9
2	17 Jan 2020	1.2	35.9	**27.2**	29.4	44.3
3	14 Jan 2020	1.7	**39.1**	43.1	48.7	45.0
4	5 Feb 2020	1.8	43.9	41.6	**38.1**	50.2
5	23 Jan 2020	1.8	38.8	31.5	**30.6**	40.0
6	9 Jan 2020	1.9	39.3	**32.8**	41.8	39.4
7	22 Jan 2020	1.9	**46.4**	46.7	49.6	49.2
8	13 Feb 2020	1.9	**38.6**	38.8	41.9	48.6
9	10 Jan 2020	2.1	42.5	**24.3**	29.1	42.9
10	29 Jan 2020	2.1	35.8	**35.2**	41.6	43.6

**Table 7:** Parameter estimates for the in-sample data, the out-of-sample data, and data generated via the MLRM with varying numbers of layers.

Code	Parameter	In-Sample	Out-of-Sample	Layer = 1	Layer = 2	Layer = 3	Layer = 4	Layer = 10
4502	κ	1.81	2.77	3.12	3.10	3.14	3.21	3.20
	γ	2.48	2.15	2.04	2.05	2.04	2.05	2.05
	ν	1.99	1.99	1.99	1.98	1.98	1.98	1.98
	α	2.70	1.30	1.22	1.12	1.06	1.05	1.05
	ν/α	0.74	1.53	1.63	1.77	1.87	1.89	1.89
6758	κ	1.74	2.67	3.19	3.52	3.63	3.65	3.65
	γ	2.53	2.24	2.02	2.02	2.00	2.01	2.01
	ν	2.54	1.99	1.99	1.99	1.98	1.98	1.98
	α	2.63	1.12	1.22	1.02	0.92	0.90	0.90
	ν/α	0.97	1.78	1.63	1.95	2.15	2.20	2.20
7203	κ	1.87	3.41	1.79	2.09	2.28	2.33	2.33
	γ	2.46	2.18	2.42	2.31	2.25	2.23	2.23
	ν	2.54	1.99	1.98	1.98	1.98	1.98	1.98
	α	2.50	0.87	1.42	1.16	1.04	1.02	1.02
	ν/α	1.02	2.29	1.40	1.71	1.90	1.95	1.95

## Data Availability

The original FLEX Full dataset was purchased from the JPX and cannot be shared due to contractual restrictions. However, the synthetic data generated in this study using the MLRM, which was trained on the JPX dataset, is available from the corresponding author upon reasonable request.
